# Expression of ST3GAL4 Leads to SLe^x^ Expression and Induces c-Met Activation and an Invasive Phenotype in Gastric Carcinoma Cells

**DOI:** 10.1371/journal.pone.0066737

**Published:** 2013-06-14

**Authors:** Catarina Gomes, Hugo Osório, Marta Teixeira Pinto, Diana Campos, Maria José Oliveira, Celso A. Reis

**Affiliations:** 1 Institute of Molecular Pathology and Immunology of the University of Porto, IPATIMUP, Porto, Portugal; 2 Instituto de Engenharia Biomédica, INEB, University of Porto, Porto, Portugal; 3 Faculty of Medicine, University of Porto, Porto, Portugal; 4 Institute of Biomedical Sciences of Abel Salazar, ICBAS, Porto, Portugal; Van Andel Institute, United States of America

## Abstract

Sialyl-Lewis X (SLe^x^) is a sialylated glycan antigen expressed on the cell surface during malignant cell transformation and is associated with cancer progression and poor prognosis. The increased expression of sialylated glycans is associated with alterations in the expression of sialyltransferases (STs). In this study we determined the capacity of ST3GAL3 and ST3GAL4 sialyltransferases to synthesize the SLe^x^ antigen in MKN45 gastric carcinoma cells and evaluated the effect of SLe^x^ overexpression in cancer cell behavior both *in vitro* and *in vivo* using the chicken chorioallantoic membrane (CAM) model. The activation of tyrosine kinase receptors and their downstream molecular targets was also addressed. Our results showed that the expression of ST3GAL4 in MKN45 gastric cancer cells leads to the synthesis of SLe^x^ antigens and to an increased invasive phenotype both *in vitro* and in the *in vivo* CAM model. Analysis of phosphorylation of tyrosine kinase receptors showed a specific increase in c-Met activation. The characterization of downstream molecular targets of c-Met activation, involved in the invasive phenotype, revealed increased phosphorylation of FAK and Src proteins and activation of Cdc42, Rac1 and RhoA GTPases. Inhibition of c-Met and Src activation abolished the observed increased cell invasive phenotype. In conclusion, the expression of ST3GAL4 leads to SLe^x^ antigen expression in gastric cancer cells which in turn induces an increased invasive phenotype through the activation of c-Met, in association with Src, FAK and Cdc42, Rac1 and RhoA GTPases activation.

## Introduction

Alterations in cell surface glycosylation are considered a hallmark during carcinogenesis. These alterations usually lead to the expression of tumor-associated carbohydrates on glycoproteins or glycolipids that decorate cell surfaces [1]. One of the most common glycan alterations is the increase of sialylated Lewis-type blood group antigens, such as sialyl Lewis A (SLe^a^ (NeuAcα2,3Galβ1-3(Fucα1-4)GlcNAc-R)) and sialyl Lewis X (SLe^x^ (NeuAcα2,3Galβ1-4(Fucα1-3)GlcNAc-R)). SLe^a^ and SLe^x^ are expressed in cancer cells, mimicking their normal expression on blood cells (monocytes and neutrophils) potentiating cancer cell migration through binding to endothelial cell selectins [2], [3]. Therefore, SLe^a^ and SLe^x^ overexpression is a common feature of several carcinomas (e.g., lung, colon, gastric and pancreas) and it is associated with increased metastatic capacity [4], [5], [6], [7] and poor patients survival [8], [9], [10], [11], [12].

The increased expression of sialylated glycans associated to carcinogenesis is the result of altered expression of sialyltransferases (STs) genes which encode for enzymes involved in the biosynthesis of the glycan antigens described above [13]. Up to 20 different sialyltransferases have been described to catalyse the transfer of sialic acid residues from a donor substrate CMP-sialic acid to the oligosaccharide side chain of the glycoconjugates. This sialic acid generally occupies the terminal non-reducing position on glycan chains [14]. Different STs show cell and tissue specific expression pattern and differ in substrate specificities and types of linkage formed [14]. Depending on these characteristics, STs are classified in four families - ST3Gal, ST6Gal, ST6GalNAc and ST8Sia. ST3Gal family are α2,3-STs which catalyze the transfer of sialic acid residues to terminal galactopyranosyl (Gal) residues and include six members from ST3Gal I to ST3Gal VI [15].

Among the six ST3Gal sialyltransferases, ST3Gal III, IV and VI have been described to contribute to SLe^x^ formation [16], [17], with a substantial role attributed to ST3Gal IV [18], [19].

The sialyl-Lewis antigens are synthesized on type 1 (Gal β1,3 GlcNAc) or type 2 (Gal β1,4 GlcNAc) disaccharide sequences. The sialyltransferase ST3Gal III preferentially acts on type 1 rather than on type 2 disaccharides and is involved in the synthesis of SLe^a^ [20]. ST3Gal IV mainly catalyzes the α2,3 sialylation of type 2 disaccharides, leading to the biosynthesis of SLe^x^ [18], [21].

We previously demonstrated the contribution of different ST3Gal sialyltransferases to the synthesis of sialyl Lewis antigens in gastric carcinoma cells, and described that ST3Gal IV is involved in the synthesis of SLe^x^ antigen [22]. In line with this report, other studies also found that high expression of ST3Gal IV, contributes to the expression of α2,3-linked sialic acid residues, and is associated with the malignant behavior of gastric cancer cells [23].

In gastric carcinoma tissues, the increased expression of ST3Gal IV [24] and of sialyl Lewis antigens have been associated with poor prognosis and metastatic capacity [8]. These reports highlight the role of STs and evidenced that the expression of crucial glycan determinants, such SLe^x^, play an important role in tumor progression. However, the molecular mechanisms underlying the aggressive behavior of gastric cancer cells expressing SLe^x^ are not fully understood. Some studies pointed to the importance of tyrosine kinase receptor activation in STs overexpression models [25], [26], [27]. In the present study we assessed the effect of ST3GAL IV overexpression in the synthesis of SLe^x^ in gastric carcinoma cells and evaluated the functional role of SLe^x^ *in vitro* (proliferation, invasion and adhesion) and *in vivo* (angiogenesis, tumor growth and invasion). We further evaluated the contribution to cell behavior of tyrosine kinase receptors activation and identified the downstream effectors in the context of ST3Gal IV/SLe^x^ overexpressing gastric carcinoma cells.

## Materials and Methods

### Cell culture

The gastric cancer cell line MKN45 was obtained from the Japanese Cancer Research Bank (Tsukuba, Japan) and was stably transfected with full length human gene for ST3GAL3 (MST3Gal III), ST3GAL4 (MST3Gal IV) and the empty vector pcDNA3.1 (Mock) as shown previously [22]. The cells were grown in monolayer culture in T75cm^2^ flasks and maintained at 37°C in an atmosphere of 5% CO_2_, in Roswell Park Memorial Institute (RPMI) 1640 GlutaMAX, HEPES medium supplemented with 10% fetal bovine serum (FBS), 1% penicillin-streptomycin (P/S) and in the presence of 0.5 mg/mL G418 (all from Invitrogen). Culture medium was replaced every two days.

### RNA isolation, cDNA synthesis and real-time PCR analysis

Total RNA was extracted from cell lysates of Mock, MST3Gal III and MST3Gal IV cell lines using TRI Reagent (Sigma) and converted to cDNA using the SuperScript® II Reverse Transcriptase (Invitrogen). Reverse transcription was performed using 3 µg of total RNA, random oligonucleotides primers and SuperScript II RT (Invitrogen) in a total volume of 20 µL as described by the manufacturer. For real-time PCR analysis, cDNA samples were diluted 50-fold with water and PCR amplified in triplicate with 10.0 µL Power SYBRGreen Master Mix (Applied Biosystems), 0.48 µL of each 10 µM primer and 4 µL cDNA using an ABI Prism 7000 Sequence Detection System (Applied Biosystems). The primers used were the following: ST3GAL3 for5'-ggtggcagtcgcaggattt-3'; rev5'-catgcgaacggtctcatagtagtg-3'; and ST3GAL4 for5'-cctggtagctttcaaggcaatg-3'; rev5'-cctttcgcacccgcttct-3'. Expression of 18S (for5'-cgccgctagaggtgaaattc-3'; rev5'-cattcttggcaaatgctttcg-3') and GAPDH (for5'-agtccctgccacactcag-3'; rev5'-tactttattgatggtacatgacaagg-3') was also measured in triplicate for each sample and used for normalization of target gene abundance. Specificity of amplification was confirmed by melting curve analysis. Standard curves were determined for each gene, and results are presented as ratio between target gene and housekeeping genes, 18S and GAPDH.

### Proliferation assays

Cell growth was analyzed using the BrdU reagent (Roche) according to the manufacturer's directions. Cells (1×10^5^) were seeded in slides on 24-well plates (Thermo Fisher Scientific) and grown in RPMI containing 10% FBS, 1% P/S in the presence of 0.5 mg/mL G418. When cells reached 50% of confluence, BrdU was incorporated in cell culture medium and incubated for 20 minutes. After incorporation cell culture medium was removed and cells fixed with methanol for 30 minutes. Cell labeling with anti-BrdU antibody and FITC secondary antibody was performed according to manufacturer's instructions. Three independent assays were performed and each assay was done in quadruplicates for all the cell lines. Percentage of dividing cells was calculated by measuring positive BrdU cells in relation to total cells with the help of ImageJ software. Results are presented as means ± SD for each sample, and proliferation levels obtained were compared with the Mock control cell line.

### Invasion assay

Invasion assays were performed in a BD Biocoat Matrigel invasion chamber with an 8-µm diameter pore size membrane and a thin layer of Matrigel, in a 24-well plate. Inserts were rehydrated for at least 1 hour in RPMI medium. After detachment of confluent cells with trypsin/EDTA, cells (5×10^4^) were seeded in the upper surface of Transwell plates and cultured in RPMI containing 10% FBS, 1% P/S in the presence of 0.5 mg/mL G418 for 6 hours, and the same culture medium was added in the lower part of the insert. After incubation, non-invading cells in the upper part of the insert were carefully removed, cells were fixed with methanol and membranes were removed from the inserts and mounted in a slide using Vectashield with DAPI (Vector labs). Three independent assays were performed and cells were seeded in duplicate for each cell line. Invading cells were counted under a fluorescence microscope, and measurement was done by counting cells in three different fields in each sample, with application of ImageJ software. Results are presented as means ± SD for each sample, and invasion levels obtained were compared with the Mock control cell line.

### Cell-substrate adhesion assay

Cell adhesion assays were performed in a 96-well plate coated overnight at 4°C with 50 µL of different extracellular matrix (ECM) proteins: collagen IV, fibronectin and vitronectin in the concentration of 20 µg/mL, while bovine serum albumin (BSA) (Sigma-Aldrich) was used as negative control. After coating, the plate was incubated for 1 hour with 0.5% of BSA in phosphate buffer saline (PBS) and viable cells (2×10^4^ cells/well) were introduced into the plate and allowed to adhere for 30 min in RPMI serum-free medium at 37°C and 5% CO_2_. Removal of non-adherent cells was performed by washing the plate with PBS and adherent cells were fixed with methanol for 30 minutes. Cells were subjected to 0.5% crystal violet dissolved in 20% of methanol for 1 hour, and then washed several times with water and allowed to air dry. Crystal violet dye was solubilized with 10% acetic acid and absorbance was measured at λ = 560 nm. Results are presented as means ± SD for each sample, and adhesion levels obtained were compared with the Mock control cell line.

### Phospho-RTK array analysis

Cells were cultured until reached confluence on T75 cm^2^ flasks with RPMI medium supplemented with 10% FBS and 100 units/mL penicillin-streptomycin in the presence of 0.5 mg/mL G418. Cells were then lysed in NP40 lysis buffer (1% NP40, 20 mM Tris-HCl (pH 8.0), 137 mM NaCl, 10% glycerol, 2 mM EDTA, 1 mM sodium orthovanadate, and protease inhibitor cocktail tablet (Roche), protein concentration was determined by the bicinchoninic acid (BCA) protein assay (Pierce) and 300 µg of total protein was used for the human Phospho-RTK array kit (R&D Systems). Phospho-RTK array protocol was performed according to manufacturer's instructions. Activated receptors were matched according to the phospho-RTK array coordinates: a1, a2: EphA6; a3, a4: EphA7; a5, a6: EphB1; a7, a8: EphB2; a9, a10: EphB4; a11, a12: EphB6; a13, a14: mouse IgG1 negative control; a15, a16: mouse IgG2A negative control; a17, a18: mouse IgG2B negative control; a19, a20: goat IgG negative control; a21, a22: PBS negative control; b1, b2: Tie-2; b3, b4: TrkA; b5, b6: TrkB; b7, b8: TrkC; b9, b10: VEGFR1; b11, b12: VEGFR2; b13, b14: VEGFR3; b15, b16: MuSK; b17, b18: EphA1; b19, b20: EphA2; b21, b22: EphA3; b23, b24: EphA4; c1, c2: Mer; c3, c4: c-Met; c5, c6: MSPR; c7, c8: PDGFRα; c9, c10: PDGFRβ; c11, c12: SCFR; c13, c14: Flt-3; c15, c16: M-CSFR; c17, c18: c-Ret; c19, c20: ROR1; c21, c22: ROR2; c23, c24: Tie-1; d1, d2: EGFR; d3, d4: ErbB2; d5, d6: ErbB3; d7, d8: ErbB4; d9, d10: FGFR1; d11, d12: FGFR2α; d13, d14: FGFR3; d15, d16: FGFR4; d17, d18: insulin R; d19, d20: IGF-IR; d21, d22: Axl; d23, d24: Dtk. Black dots represent phospho-tyrosine positive controls.

### c-Met and Src inhibition assay

c-Met and Src inhibitors were used to evaluate the invasive capacity of the cells upon inhibition. c-Met inhibition was performed with 0.1 µM of PHA-665752 (Sigma) and Src inhibition with 20 µM of PP2 (Sigma) both during 10h. Inhibition was assessed by Western blot for the phosphorylation status of c-Met and Src, and invasion capacity of cells was evaluated as described above, after 10h of inhibitors incubation.

### Immunoblotting

Proteins were obtained from total cell lysates of each cell line. Briefly, confluent T75 cm^2^ flasks were incubated with NP40 lysis buffer and cells were scraped. Total cell lysates were centrifuge at 14000 rpm for 10 minutes to remove pellet cell debris. Protein concentration was determined by the bicinchoninic acid (BCA) protein assay (Pierce). Proteins from cell lysates were separated accordingly to protein molecular weight by gel electrophoresis in 7.5% acrylamide/bis acrylamide (Sigma) SDS-PAGE. For c-Met, phospho-Met, phospho-AKT, phospho-STAT3 and phospho-ERK detection, 25 µg of total protein extract were used and for phospho-Src and phospho-FAK detection we used 50 µg of total protein extract. Gels were then transferred onto a nitrocellulose membrane (Amersham) in a semi-dry system. Membranes were then blocked with 5% non-fat milk, washed three times with Tris buffer saline (TBS), and incubated overnight at 4°C with primary antibodies. After incubation, membranes were washed three times with TBS and incubated 1 hour with secondary antibodies. Analysis was done by chemiluminescence using the ECL Western blotting detection reagent and films (both from GE Healthcare).

Antibodies: anti-phosphorylated Akt Ser473, anti-phosphorylated FAK Tyr397, anti-phosphorylated Src Tyr416, anti-phosphorylated ERK Thr202/Tyr204 and anti-phosphorylated MET Tyr1234/1235, anti- phosphorylated STAT3 Tyr705 (all rabbit polyclonal antibody from Cell Signaling Technology) were used at 1∶1000 dilution. Mouse monoclonal IgG2a antibody directed against human MET (Invitrogen) was used at 1∶2000. Anti-SLe^x^ clone KM93 (Millipore) was used at 1∶500 dilution. Goat anti-actin and rabbit anti-actin (Santa Cruz Biotechnology) were used at 1∶8000 dilution. Secondary anti-rabbit and anti-goat antibodies, conjugated with horseradish peroxidase (DAKO), were used at 1∶2000, while anti-mouse IgG2a and IgM antibodies, conjugated with horseradish peroxidase (Jackson immunoresearch) were used at 1∶25000 and 1∶10000, respectively.

### Cdc42, Rac1 and RhoA GTPases pull down assay

Cells were cultured in serum free medium for 24 hours, and proteins were obtained from total cell lysates. Pull-down assays, using RhoA/Rac1/Cdc42 Activation Assay Combo Biochem Kit (Cytoskeleton, inc), were performed according to manufacturer’s instructions, using 600 µg of total protein lysates. Briefly, rhotekin-RBD effector domain affinity beads were used to bind RhoA active (GTP-bound) protein and PAK-PBD effector domain affinity beads for Cdc42 and Rac1 active proteins. Total proteins were incubated with these beads for 2 hours, and pull down proteins were eluted with Laemmli buffer and separated on 12% acrilamide/bis acrilamide gels. Pull down negative and positive controls were performed according to manufacturer’s instructions; briefly total cell lysates were incubated with GTPases inhibitors or activators prior to pull down. To confirm the presence of GTPases in the cell protein extract, a 5% input control were also runned in the gels. Gels were transferred to nitrocellulose membranes and antibodies against Cdc42, RAC1 and RhoA (included in the kit) were incubated overnight with gentle agitation. Proteins were analyzed by chemiluminescence using the ECL Western blotting detection reagent and films (both from GE Healthcare).

### Chicken embryo *in vivo* tumorigenesis and angiogenic assay

The chicken embryo chorioallantoic membrane (CAM) model was used to evaluate the angiogenic response and growth capability of Mock and MST3Gal IV cells (n = 13 for each group). According to the European Directive 2010/63/EU, ethical approval is not required for experiments using embryonic chicken. Correspondingly, the Portuguese law on animal welfare does not restrict the use of chicken eggs. Briefly, fertilized chick (*Gallus gallus*) eggs obtained from commercial sources were incubated horizontally at 37.8°C in a humidified atmosphere and referred to embryonic day (E). On E3 a square window was opened in the shell after removal of 1.5–2 mL of albumin to allow detachment of the developing CAM. The window was sealed with a transparent adhesive tape and the eggs returned to the incubator. The window in the egg shell does not interfere in any way with the normal development of the chick embryo. Cells, re-suspended in 10 µL of complete medium (1×10^6^ cells per embryo), were placed on top of E10 growing CAM into a 3 mm silicon ring under sterile conditions. The eggs were re-sealed and returned to the incubator for an additional 3 days. At this point the embryos are at E 13, thus still in the first 2/3 of development. The embryos were euthanized by adding 2 mL of fixative in the top of the CAM which is a very efficient and fast method. After removing the ring, the CAM was excised from the embryos, photographed *ex ovo* under a stereoscope, at 20x magnification (Olympus, SZX16 coupled with a DP71 camera). The number of new vessels (less than 20 µm diameter) growing radial towards the ring area was counted in a blind fashion manner. The area of CAM tumors was determined using the Cell A (Olympus) software.

### Immunohistochemistry analysis and tumor invasive phenotype

Excided CAMs were fixed in 10% neutral-buffered formalin, paraffin-embedded for slide sections and stained with hematoxilin-eosin for histological examination. Slides with clear view of the CAM tumors were also processed for cytokeratin, SLe^x^ and p-Met immunohistochemical detection in order to characterize the phenotype of CAM tumors. Briefly, sections were dewaxed, rehydrate and the endogenous peroxidase activity was blocked with 3% H_2_O_2_ in methanol for 30 minutes. Then, sections were incubated with normal rabbit or swine serum diluted 1∶5 in PBS containing 10% BSA for 30 minutes followed by incubation with the monoclonal antibodies overnight at 4°C. Incubation with both biotinylated rabbit anti-mouse and swine anti-rabbit secondary antibodies (DAKO) was done during 30 minutes at room temperature followed by avidin/biotin complex detection (Vectastain). Staining was performed with 3,3′-diaminobenzidine tetrahydrochloride (Sigma) containing 0.02% hydrogen peroxide and counter staining of the nucleus was done with Mayer’s hematoxylin. Monoclonal antibodies used were KM93 1:60, p-Met 1∶100 and cytokeratins AE1/AE3 1∶300, and for both antigen retrieval was achieved with citrate buffer pH∶6.0. Evaluation of tumour invasion was performed in a blind fashion way by two independent observers. The semi-quantitative evaluation took into consideration the quantity of human AE1/AE3 labeled cells present in the CAM mesenchyme.

### Statistical analysis

Statistical analysis was performed using Graph Pad program. ANOVA tests were used to calculate significance in an interval of 95% confidence level. All statistics were compared with Mock group and values of p<0.05 were considered to be statistically significant.

## Results

### Induction of SLe^x^ by overexpression of ST3GAL4 in gastric carcinoma cells

**Figure 1:**
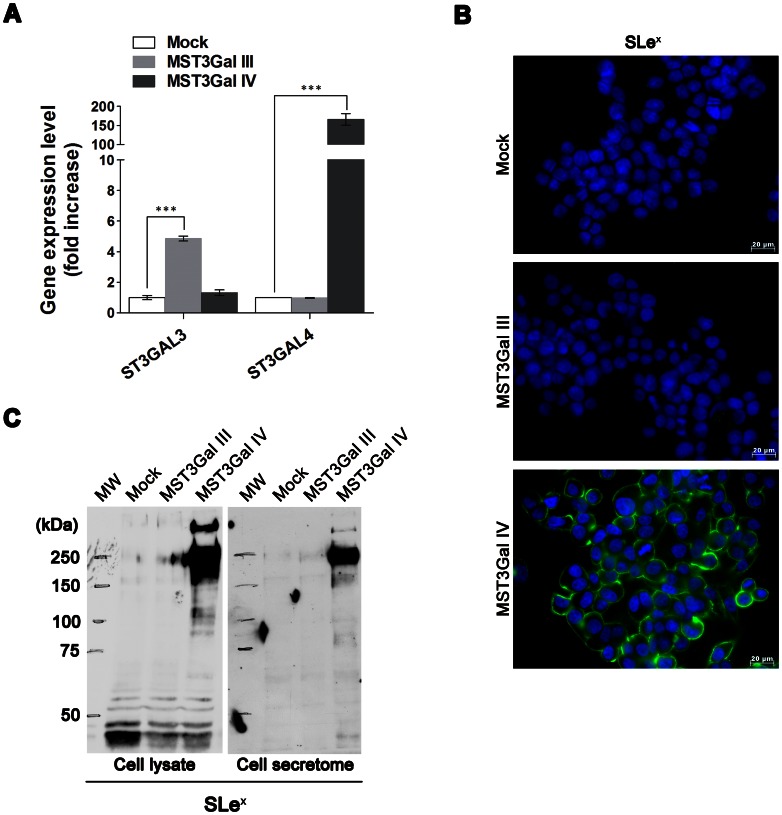
Induction of SLe^x^ expression by ST3Gal IV transfection in gastric carcinoma cells. **A** – Relative quantification of ST3GAL3 and ST3GAL4 mRNA expression in MOCK, MST3Gal III and MST3Gal IV transfected cells showing a significant overexpression of ST3GAL3 gene in MST3Gal III cells and ST3GAL4 in MST3Gal IV cells when comparing gene expression levels in mock cells; ***p<0.001 Mock versus MST3Gal III for ST3GAL3 gene and ***p<0.001 MST3Gal IV versus Mock for ST3GAL4 gene. Results are presented as means ± SD. **B** – Immunofluorescence detection of SLe^x^ expression in Mock, MST3Gal III and MST3Gal IV cells evidencing the presence of SLe^x^ in MST3Gal IV (magnification 200x); **C** – Western blot detection of SLe^X^ in proteins from total cell lysate and secreted proteins from Mock, MST3Gal III and MST3Gal IV cells. SLe^x^ expression was observed on cell lysates and secretome from ST3GAL4 transfected cells (MST3Gal IV).

To evaluate the role of ST3GAL3 and ST3GAL4 sialyltransferases in the synthesis of SLe^x^ structures, the previously established MKN45 cell line model stably transfected with full length of either ST3GAL3 (MST3Gal III), ST3GAL4 (MST3Gal IV) genes, or an empty vector as control (Mock) were used [22]. The evaluation of the expression levels of ST3GAL3 and ST3GAL4 genes by Real Time-PCR (Figure 1A), showed approximately 4 fold increase of ST3GAL3 gene in MST3Gal III cells in comparison with Mock and MST3Gal IV cells, and a 160 fold increase of ST3GAL4 gene expression in MST3Gal IV cells in comparison with Mock and MST3Gal III cells.

The biosynthesis of SLe^x^ antigen was further assessed by immunofluorescence and by Western blot analysis of total cell lysates and secreted proteins (secretome). Immunofluorescence results showed expression of SLe^x^ in MST3Gal IV cells when compared with Mock and MST3Gal III cell lines (Figure 1B). Consistently, Western blot results demonstrated the expression of SLe^x^ in MST3Gal IV cells, both in total cell lysates as previously described [22] and secreted proteins (Figure 1C). No expression of SLe^x^ was detected in total protein extracts or secreted proteins from Mock and MST3Gal III cells.

Since only MST3Gal IV transfected cells were able to produce SLe^x^ antigen, further experiments were performed using the MST3Gal IV and Mock cells.

### *In vitro* biological behavior of SLe^x^ expressing cells -MST3Gal IV

**Figure 2:**
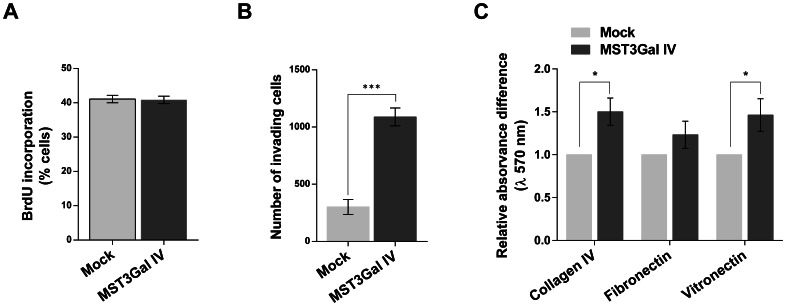
SLe^x^ overexpression induces cell invasion and increases matrix-cell adhesion *in vitro*. **A** - BrdU proliferation assay in transfected cells. After 50% confluence, cells were incubated for 30 min with BrdU reagent and fluorescent labeled for proliferative index measurement. No differences were found between the different cell lines. **B** - Cell invasion assay on Matrigel chambers. Cells were seeded on Matrigel-coated filters inserted into two-compartment chambers and invading capacity was measured by counting the number of cells that invade, through the Matrigel-coated filter, 6 hour after incubation. MST3Gal IV cells demonstrated an invasive phenotype presenting a significant increased number of invasive cells when compared with MOCK cells (***p < 0.001). **C** - Cell adhesion to ECM proteins. Adhesion potential of cells was assessed by incubating cells 30 minutes in pre-coated plates with collagen IV, fibronectin and vitronectin. Results demonstrate an increased adhesion of SLe^x^ expressing cells to collagen IV (*p < 0.05 MST3Gal IV versus Mock) and vitronectin (*p < 0.05 MST3Gal IV versus Mock) matrix proteins. No statistical difference was found in the adhesion capacity of cells to fibronectin. Results are presented as means ± SD.

Cell growth, invasive capacity and adhesion properties of cells transfected with the ST3Gal IV gene were evaluated in order to characterize these cellular phenotypes, and also to address the biological role of SLe^x^ expression. MST3Gal IV cells showed no statistical differences when compared to Mock cells in terms of BrdU incorporation (Figure 2A), suggesting that the expression of ST3Gal IV sialyltransferase and of SLe^x^ do not affect the proliferation rate of these cells.

Cell invasion was analyzed by counting the number of invasive cells on Transwell Matrigel invasion chambers. This analysis revealed that cells overexpressing ST3Gal IV sialyltransferase presented 3 fold increased ability to invade *in vitro,* when compared with Mock control cells (Figure 2B). This result evidence the importance of SLe^x^ expression for the invasive phenotype of MST3Gal IV cells.

In addition, the adhesion to extracellular matrix proteins was also evaluated by seeding cells in plates pre-coated with collagen type IV, fibronectin or vitronectin. Interestingly, SLe^x^ expressing cells (MST3Gal IV) present an increase capacity to adhere to collagen IV and to vitronectin when compared with Mock control cells (Figure 2C). In contrast, no statistical differences were found in the adhesion capacity of these cells to fibronectin.

### *In vivo* evaluation of angiogenesis, tumor growth and invasion capacity of MST3Gal IV cell line using chicken embryo chorioallantoic membrane model

Transfected cells were inoculated into the chicken embryo chorioallantoic membrane (CAM) and different parameters were evaluated after 3 days of inoculation, specifically, the angiogenenic response, tumor size and tumor cell invasive capacity (Table 1). The angiogenic potential was assessed by counting the number of vessels with less than 20 µm diameter growing radially towards the inoculation area. The results show no statistical differences in vessel number between Mock control cells and MST3Gal IV indicating that ST3Gal IV and SLe^x^ expression do not influence the angiogenic response. Tumor size was assessed by measuring the area (mm^2^) of the tumor in the different groups. The results show no statistical differences in tumor size arising from the different cell lines, indicating no influence of ST3Gal IV and SLe^x^ expression in tumor growth potential.

**Table 1:** Parameters evaluated in the CAM model: angiogenesis, tumor growth and invasion potential of cells.

	MOCK	MST3Gal IV	p-value
**Angiogenesis** ***(vessel number ± SEM)***	20.15±4.06	19.31±4.05	n.s.
**Tumour growth** ***(total area mm ^2^± SEM)***	4.13±1.45	4.29±1.24	n.s.
**Cell invasion on CAM** ***(% cases)***	1/714.3%	5/771.43%	*0.0308

n.s.(non-significant) p≥0.05.

* p<0.05.

SEM Standard Error of the Mean.

**Figure 3:**
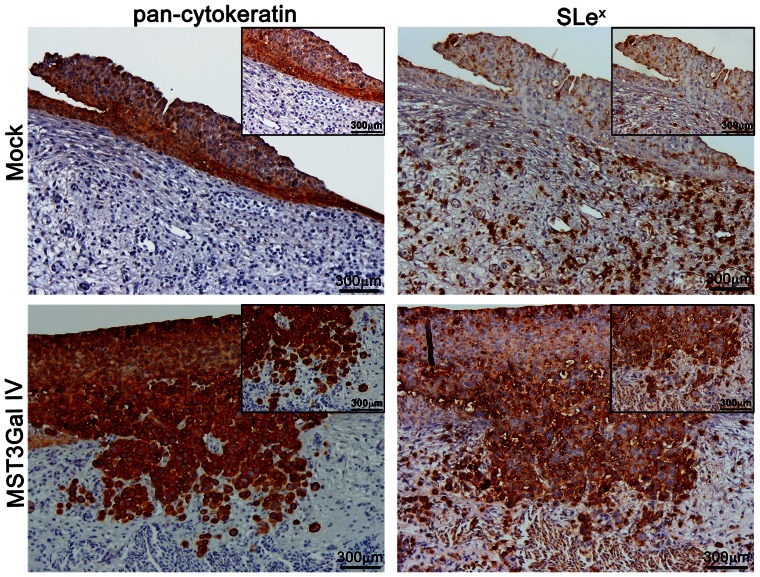
SLex expressing cells present an invasive phenotype *in vivo* chicken embryo chorioallantoic membrane (CAM) model. Transfected cells were inoculated in CAM and different parameters were evaluated after 3 days of inoculation. Invasive capacity of inoculated cells was evaluated by immunolabeling CAM tumors with human cytokeratin and SLe^x^ to assess the presence of human epithelial cells expressing SLe^x^. Human cytokeratins immunostaining was used to prove the presence of inoculated human gastric carcinoma cells. SLe^x^ expression and the invasive capacity of cells were match up to cytokeratins expression. Immunostained tissues evidence the presence of SLe^x^ structures in CAM tumors from MST3Gal IV cells that, in constrast with Mock cells, invaded the CAM tissue.

For the evaluation of tumor cell invasive capacity, CAMs were excised from the embryos, fixed with formalin and paraffin-embedded. Invasion of inoculated cells was evaluated in sections of CAM tumors immunostained for human citokeratins. The results show an increased invasive capacity of MST3Gal IV cells inoculated in CAM in comparison to Mock cells (Table 1). To assess if cells invading the CAM expressed SLe^x^ antigens, CAM sections were immunostained for SLe^x^. The results show that MST3Gal IV invasive cells expressed SLe^x^ antigens, contrary to the observed in Mock control cells (Figure 3).

### Increased activation of c-Met receptor in SLe^x^ expressing cells-MST3GalIV

**Figure 4:**
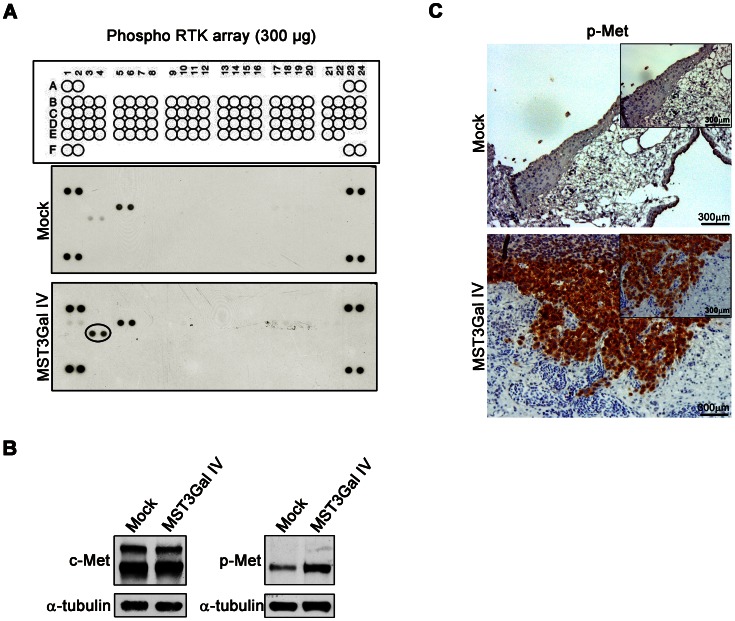
Tyrosine kinase receptors activation evaluation in gastric carcinoma cells; increased c-Met in MST3GAL IV cells. **A** – phospho-RTK array of transfected Mock and MST3Gal IV cells. Total cell lysates were collected and 300 µg of total protein were incubated into a phospho-RTK membrane array. The array shows an increased activation of c-Met (HGFR) in MST3Gal IV cells. Activated receptors were matched according to the phospho-RTK array coordinates indicated in the material and methods section. c3, c4 correspond to c-Met ; **B** – Analysis of c-Met activation in MOCK and MST3Gal IV cells by Western blot; Cell lysates were analyzed by Western blot with antibodies directed against human c-Met and the phosphorylated tyrosine residues 1234/1235 of the kinase domain to confirm the activation of c-Met in MST3GAL IV cells. The results show an increase expression of phosphorylated c-Met (p-Met) in MST3Gal IV cell line with no differences in c-Met total protein levels in both cell lines. Anti-tubulin antibody was used to assess loading. **C** – Expression of phosphorylated c-Met was assessed in CAM tissues. The evaluation of c-Met activation in CAM tumors show that MST3Gal IV/SLe^x^ expressing cells present positive staining for phospho c-Met and that the resulting invading cells are also positive for the phosphorylated form of this receptor.

To evaluate the possible effects of SLe^x^ expression on the activation of cell surface receptors and on the induction of the cancer cell invasive phenotype, a receptor tyrosine kinase array was performed using total cell lysates from Mock and MST3Gal IV cells. The results show consistently that MST3Gal IV cells induce increased activation of hepatocyte growth factor receptor (HGFR/c-Met) (Figure 4A). The increased level of c-Met receptor tyrosine phosphorylation (p-Met) was further evaluated by Western blot, and the results confirmed that phosphorylation of c-Met is increased in MST3Gal IV cells, with no differences in total c-Met protein expression levels (Figure 4B).

To assess if the CAM invading cells are expressing the activated c-Met, CAM sections were immunostained for phospho c-Met, demonstrating that MST3Gal IV invasive cells are indeed expressing activated c-Met (Figure 4C).

### Evaluation of downstream effectors of c-Met activation

**Figure 5:**
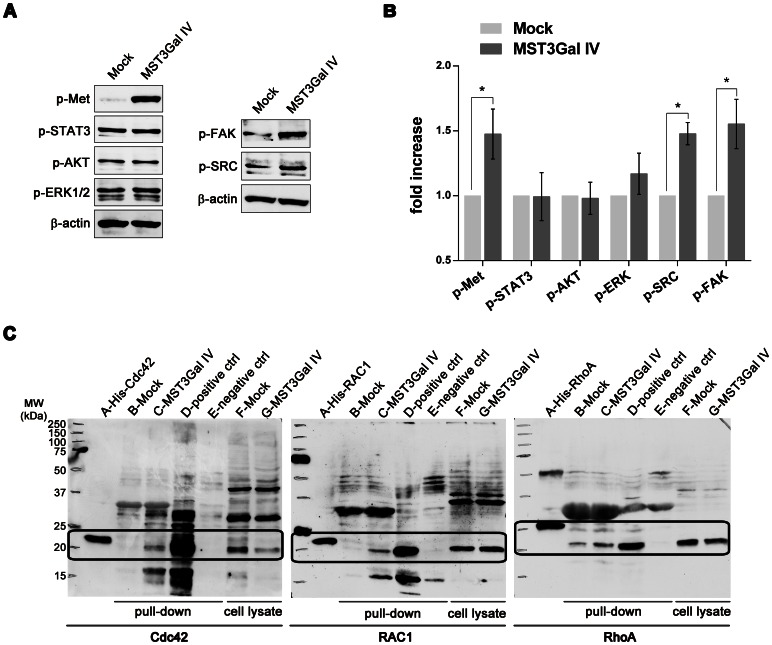
Evaluation of downstream effectors of c-Met activation. **A** – Increased levels of p-FAK and p-Src proteins in MST3Gal IV cells. The contribution of other effector proteins, such as AKT, ERK, FAK and Src was evaluated by Western blot for their phosphorylated forms in Mock and MST3Gal IV cell lines, and expression of β-actin protein was used as protein loading control. Results show increased levels of phophorylated FAK and Src supporting their involvement as downstream effectors of phosphorylated c-Met (p-Met). **B** – Analysis of 5 independent Western blot of c-Met, STAT3, AKT, ERK, FAK and Src phosphorylated forms in MOCK and MST3 Gal IV cells showing statistically significant increased levels of p-FAK and p-Src, concomitantly with increased phosphorylated c-Met. Results are presented as means ± SD. **C** - Evaluation of Cdc42, Rac1 and RhoA GTPases as potential modulators of c-Met activation by pull-down assay of their activated forms. Western blot analysis of pull-down proteins evidence an increased activation of Cdc42, Rac1 and RhoA in MST3Gal IV cell line. A-GTPase WB protein positive control (His-Cdc42, His-Rac1 and His-RhoA); B-Mock total cell protein pull down; C-MST3Gal IV total cell protein pull down; D-Mock total cell protein pull down with previous GTPases activation (pull down positive control); E-Mock total cell protein pull down with previous GTPases inhibitors (pull down negative control); F-Mock total cell protein input; G-MST3Gal IV total cell protein input. Highlighted areas represent regions of interest regarding the specific protein migration.

c-Met activation relies on stereotypical signaling modulators common to many RTKs [28], [29]. To evaluate possible downstream effectors of c-Met activation, we analyzed the activation of Src, FAK, STAT3, AKT and ERK, proteins involved in different c-Met downstream pathways. Our results show that MST3Gal IV cells present increased activation of Src and FAK proteins which are known to be involved in cell motility and invasion (Figure 5A, B). The small GTPases of the Rho family, such as Rac1, Cdc42, and RhoA were also evaluated as possible downstream modulators of c-Met activation by pull-down of activated GTPases. Our results demonstrate that in MST3Gal IV cells, the expression of sialyltransferase IV and SLe^x^ induce activation of Rac1, Cdc42 and RhoA (Figure 5C)**.**

### Inhibition of invasion in SLe^x^ expressing cells using c-Met and Src activation inhibitors

**Figure 6:**
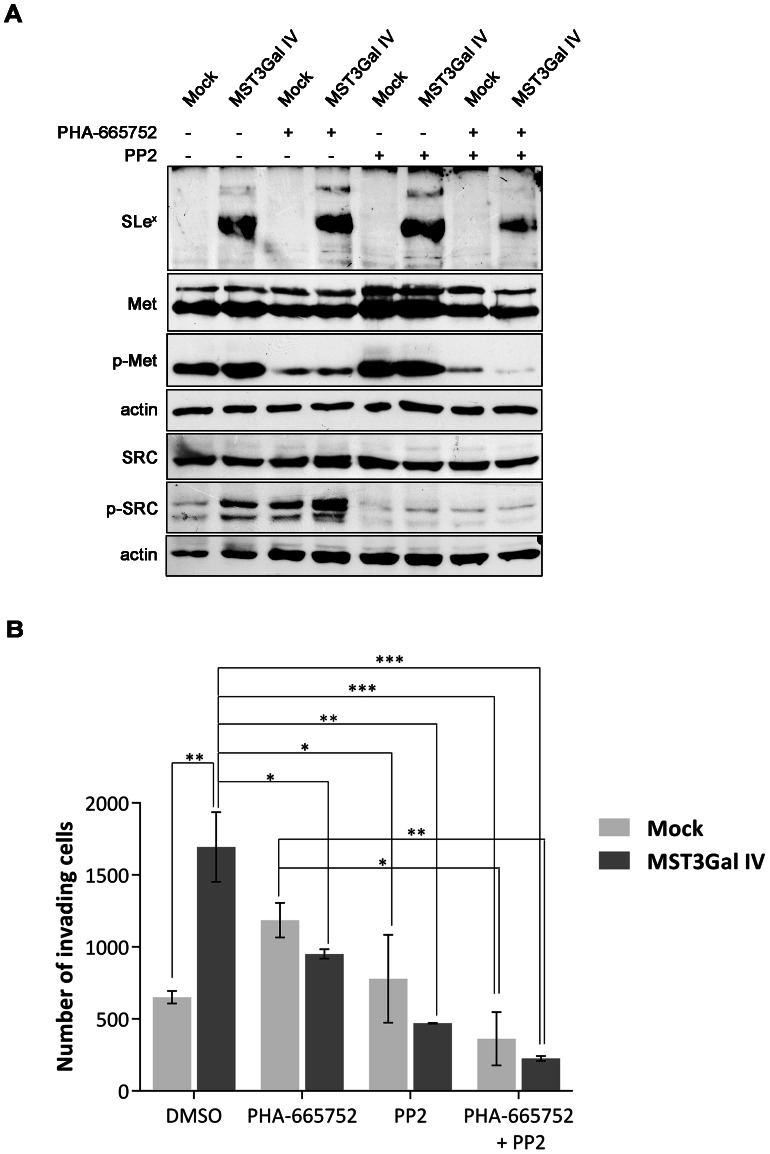
Inhibition of invasion in SLex expressing cells by targeting c-Met and Src activation. **A -** Evaluation by WB of the activation of c-Met and Src in the MST3Gal IV and Mock cells with or without the presence of inhibitors of c-Met (PHA-665752) and Src (PP2). **B -** Cell invasion assay on Matrigel chambers after inhibition of c-Met and Src activation. Cells were seeded on Matrigel-coated filters after incubation with inhibitors for 10 hours. Invading capacity was measured by counting the number of cells that invade, through the Matrigel-coated filter, after 6 hours. MST3Gal IV cells demonstrated an invasive phenotype presenting a significant increased number of invasive cells when compared with MOCK cells. Inhibition of c-Met, Src, and both in combination led to abolishment of cell invasion capability of the MST3Gal IV cells (*p<0.05; **p<0.01; ***p < 0.001).

In order to confirm the biological role of c-Met and Src activation in the invasive capacity of SLe^x^ expressing cells, inhibition of phosphorylation of c-Met, Src and both in combination were performed. The inhibition was tested using different concentration of each inhibitor, and different time-points of incubation (data not shown). Longer incubations with 0.1 µM of PHA-665752 c-Met inhibitor (24h and 48h) led to decrease in cell proliferation and cell death (data not shown), therefore a 10h incubation time-point, showing no alteration in cell proliferation, was used for the evaluation of cell invasion. Src inhibition occurred after 10h of incubation with 20 µM of PP2 and no differences in cell death and proliferation was observed after longer incubation periods (data not shown). The activation status of c-Met and Src was assessed by Western blot analysis, and results confirmed the decreased in activation of both proteins after 10 h incubation with the inhibitors (Figure 6A).

Given the observation that SLe^x^ expressing cells present increased cell invasive capacity resulting from the activation of c-Met and Src, invasion of cells was evaluated after c-Met and Src inhibition. The results confirmed the increased invasion of SLe^x^ expressing cells in DMSO control treatment, and demonstrated the abolishment of this invasion capacity upon inhibition of c-Met, Src and both proteins in combination (Figure 6B). Moreover, the results showed that inhibition of Src or both Src and c-Met in combination were more effective in precluding cell invasion (Figure 6B).

## Discussion

Aberrant glycosylation has been described for many years as a hallmark of cancer, and many of the resulting altered glycosyl epitopes are tumor associated antigens [30], [31]. These cancer-related antigens are caused by disease-specific alterations in the glycan synthesis pathway such as changes in the Golgi and Endoplasmic Reticulum compartments, mutations in enzymes or chaperons, altered expression of enzymes and biochemical competition, and even variations in sugar donor availability [for a review see 1]. A common alteration is the abnormal expression of sialyltransferases, responsible for adding sialic acids residues to cell surface molecules and to secreted proteins, and which have been involved in the oncogenic transformation, as well as in invasion and metastasis [30], [31], [32]. Sialic acids are typically attached to the outermost ends of glycoproteins and glycolipids that can mediate and modulate a wide variety of physiological and pathological processes [33].

The SLe^x^ antigen is a sialylated glycan structure which expression has been associated with cancer progression and aggressiveness as well as poor overall patient survival [4], [5], [6], [7], [8], [9], [10], [11], [12]. The expression of SLe^x^ in cancer results from the altered expression of sialyltransferases, that adds the sialic acid in a α2,3 linkage to Galactose residues on type-II chains [13].

In this study, we have characterized the role ST3Gal IV sialyltransferase in the synthesis of SLe^x^ antigen. Expression analyzes of SLe^x^ in stably transfected gastric carcinoma cells by immunofluorescence and Western blot confirmed that ST3Gal IV leads to the biosynthesis of SLe^x^. Moreover, our results indicate that SLe^x^ antigen is expressed on proteins from total cell lysates as well as on secreted proteins from MST3Gal IV cells. These results confirm previous observations that described the importance of ST3Gal IV in the synthesis of SLe^x^, the glycan ligand of selectins [18], [19]. In addition, our results are in agreement with recent reports showing an increased mRNA level of ST3Gal IV and α2,3 sialic acid residues expression in gastric cancer tissues [23].

The carbohydrate SLe^x^ functions as a ligand for cell adhesion molecules of the selectin family, usually expressed on vascular endothelial cells. The expression of SLe^x^ on cancer cells is known to facilitate tumor cell spreading by mediating tumor-endothelial cell interactions [34], [35]. The SLe^x^ antigen is known to be important in selectin interactions participating in the adhesion of cancer cells to vascular endothelium thus contributing to hematogenous metastasis [36]. These previous observations further support that SLe^x^ antigen plays a functional role in malignant cancer cell behavior. Noteworthy, the crosstalk between cancer cells and host mechanisms like cell-cell adhesion and cell-matrix adhesion interactions, tumor cell growth and motility are known to be important in modulating the process of cancer cell invasion. In the present study we performed a comprehensive evaluation of the biological role of SLe^x^ in gastric cancer cells using *in vitro* and *in vivo* models. The *in vitro* analysis showed that SLe^x^ expressing cells display a similar proliferative rate when compared with Mock transfected cells. However, SLe^x^ expressing cells demonstrated a higher capacity to invade *in vitro* in Matrigel chambers, demonstrating the active role of this sialylated glycan structure in tumor cell motility and invasion. Concomitant to this invasive capacity, SLe^x^ expressing cells evidenced higher capacity to adhere to collagen IV and vitronectin extracellular matrix proteins. These findings highlight the importance of this sialylated glycan in the malignant invasive phenotype. Furthermore, this invasive phenotype was also confirmed *in vivo* where cells transfected with ST3Gal IV and expressing SLe^x^ antigen presented increased capacity to invade the chorioallantoic membrane of the chicken embryo. Our results are in keeping with studies that associate SLe^x^ expressing tumors with more aggressive phenotypes [8], [37], [38]. In the gastric carcinoma context it has also been described that SLe^x^ antigen expression correlates with liver metastasis [7]. The modulation of cancer cell biological behavior by sialylated glycans has been previously described in human pancreatic cells. In this pancreatic model the restoration of α1,2 fucosyltransferase activity, a enzymatic competitor of ST3Gal transferases, reduces the expression of Sialyl Lewis antigens and decreases the adhesive and metastatic properties of these cells [39].

In addition, previous reports have shown that increased cellular sialylation leads to receptor and signaling pathways activation and that the hypersialylation contributes to cancer progression and increased cell motility [40], [41]. Moreover, it has been described that TNF-α can induce SLe^x^ and 6-sulfo-SLe^x^ expression in human cancer cells, by increasing the expression of ST3GAL4 [21]. This mechanism has also been shown to be mediated by neutrophils expressing TNF-α leading to cancer cells invasiveness and metastasis [42].

In order to clarify the potential implication of ST3Gal IV and of its product SLe^x^ in the biological behavior of gastric cancer cells, we evaluated the expression of activated tyrosine kinase receptors and downstream modulators involved in cancer cell invasion. The tyrosine kinase receptor array allowed the identification of a constitutive activation of c-Met in SLe^x^ expressing cells. The activation of tyrosine receptors, directly or indirectly by glycan antigens has previously been observed in other cancer cell models. Singh and colleagues described that the Thomsen-Friedenreich antigen (T antigen) present in CD44v6 promotes the activation of c-Met and MAPK signaling leading to cancer cell proliferation [43]. Furthermore, activation of c-Met receptor has been described in a breast cancer cell model that overexpress glycosyltransferases and this activation has been implicated in proliferation and invasion of cancer cells [25], [26], [27]. The MKN45 cell line model has been reported to have a high level of expression and dependence on c-Met [44] and therefore modulation of cellular glycosylation can have implications in this c-Met dependent cells.

c-Met overexpression has been considered a hallmark of cancer, playing a role in many tumors and in metastatic progression [45]. In gastric cancer, c-Met expression alterations have been reported, such as the Tpr/Met rearrangement [46], [47] and c-Met copy number amplification [48], as well as increased c-Met activation [49], [50]. We evaluated the c-Met activation in a series of gastric carcinoma tissues (data not shown). The variability in tissue sample processing is known to lead to loss of protein phosphorylation which precluded the detection of phosphorylated c-Met in these samples. However, sections analyzed from the chicken chorioallantoic membrane tumors, derived from either Mock or SLe^x^ expressing gastric carcinoma cells, confirmed phosphorylated c-Met positive staining in SLe^x^ cancer invading cells. This result further supports the hypothesis that SLe^x^ expressing cells exhibit invasive capacity through the activation of c-Met.

The activation of c-Met is well known to induce docking sites for proteins that mediate downstream signaling leading to the activation of the mitogen-activated protein kinase (MAPK), phosphatidylinositol 3-kinase (PI3K)-AKT, v-src oncogene homolog (Src), signal transducer and activator of transcription (STAT), which are signaling pathways that are involved in increased cell growth, scattering, motility, invasion, protection from apoptosis, branching morphogenesis, and angiogenesis [28], [29]. Taking that into consideration, we evaluated the downstream effectors of c-Met activation and found that FAK and Src proteins showed increased activation in cells expressing ST3Gal IV. In combination with our invasion assays results (*in vitro* and *in vivo*), these results strongly suggest that c-Met activation mediates tumor cell motility and invasion, also in gastric cancer cells. These results are in agreement with previous studies that associate Src-FAK signaling pathway with the metastization process [51], [52], [53], [54]. Furthermore, our results show that inhibition of c-Met and Src could preclude the increased invasion observed in SLe^x^ expressing cells supporting the importance of this glycosylation alteration in the activation of this invasive related pathways.

**Figure 7:**
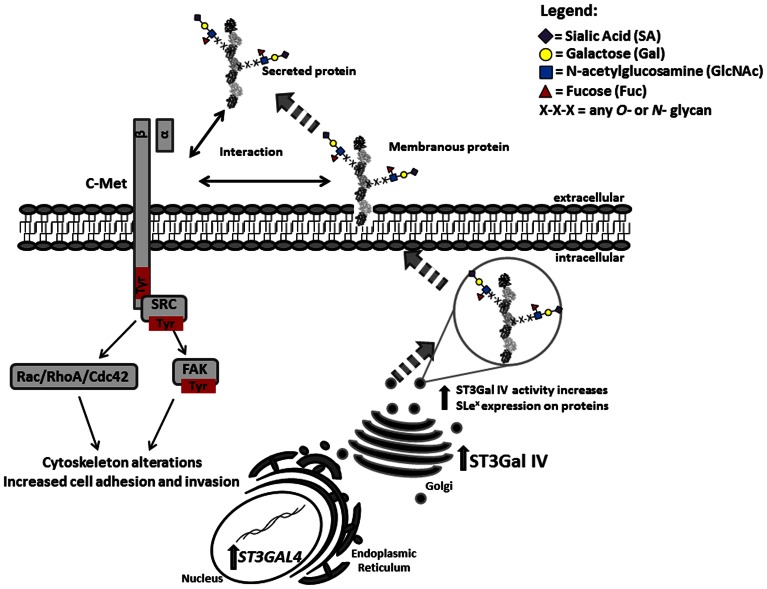
Schematic representation of the alterations induced by increased expression of SLe^x^ and activation of c-Met. Increased transcription of ST3GAL 4 leads to increased expression of the ST3Gal IV enzyme in the Golgi apparatus of the cells. This enzyme will glycosylate type-2 terminal oligosaccharide chains leading to the presence of SLe^x^ in glycoproteins targeted for the membrane or to be secreted by the cells. The expression of SLe^x^ in membrane-associated and secreted proteins can promote the interactions between these proteins and c-Met leading to its activation. c-Met activation leads to downstream signaling activation target proteins Src, FAK and Rho GTPAses leading to a modified cell-matrix adhesion and an increased cell invasion.

Oncogenic transformation is often associated with changes in organization of the cytoskeleton, which can influence cell migration, adhesion and invasion. The c-Met activation can cause changes in gene expression of cell-cycle regulators (Cdk6, p27), extracellular matrix proteinases (such as matrix metalloproteinases and urokinase plasminogen activator), and in alterations of cytoskeleton functions that control migration, invasion and proliferation [55]. The cytoskeleton is composed of a complex and organized network of various fibrous proteins within the cytoplasm, playing an essential structural and regulatory role in the maintenance of cell structure and strength, in cell division, proliferation, motility, invasion and also in signaling functions [56], [57], [58]. The activation of tyrosine kinase receptors can modify the phosphorylation status of cytoskeleton regulatory and structural proteins. Signaling pathways initiated by the activation of cell surface receptors can promote distinct membrane protrusions by converging onto the Rho family of GTPases [59], [60]. Rho proteins are small (21-25 kDa) molecules that share structural homology and become activated only when bound to GTP. One of the best characterized Rho GTPase family members is RhoA regulating the formation of stress fibers and focal adhesion assembly, while Rac1 and Cdc 42 are mainly involved in membrane ruffling and formation of filopodia, respectively [61]. Estimation of GTPases activation is frequently a molecular marker in the evaluation of cytoskeleton alterations during cell migration [62], [63], [64]. Here we showed the activation of Rho GTPases, specifically RhoA, Rac1 and Cdc42. These results further supports the evidence that SLe^x^ expression leads to cytoskeleton protein alterations in cancer cells, underlying the observed increased cell motility and invasion of these cells. Our findings are in keeping with previous reports showing the importance of RhoA, Rac1 and Cdc42 in cancer progression [65], and also the crosstalk between these GTPases and other signaling pathways like Src-FAK in the migratory phenotype of cancer cells [66]. Our present findings support the hypothesis that increased expression of SLe^x^ on the surface of malignant cells plays an important role in tumor invasion and metastasis. Overall, our study showed that tumor cell invasion is induced by SLe^x^ expression on gastric cancer cells through the activation of c-Met in association with downstream signaling effectors Src, FAK and RhoA GTPases activation (Figure 7).

These results open new avenues to design intervention strategies that target ST3Gal IV/SLe^x^ in cancer cells as well as the inhibition of c-Met and Src in order to improve gastric cancer treatment by targeting invasion and metastasis.

## References

[1] Reis CA, Osorio H, Silva L, Gomes C, David L (2010) Alterations in glycosylation as biomarkers for cancer detection. J Clin Pathol 63: 322–329. 20354203 10.1136/jcp.2009.071035

[2] Fuster MM, Esko JD (2005) The sweet and sour of cancer: glycans as novel therapeutic targets. Nat Rev Cancer 5: 526–542. 16069816 10.1038/nrc1649

[3] Varki A (1994) Selectin ligands. Proc Natl Acad Sci U S A 91: 7390–7397. 7519775 10.1073/pnas.91.16.7390 PMC44407

[4] Borsig L, Wong R, Hynes RO, Varki NM, Varki A (2002) Synergistic effects of L- and P-selectin in facilitating tumor metastasis can involve non-mucin ligands and implicate leukocytes as enhancers of metastasis. Proc Natl Acad Sci U S A 99: 2193–2198. 11854515 10.1073/pnas.261704098 PMC122341

[5] Fukuoka K, Narita N, Saijo N (1998) Increased expression of sialyl Lewis(x) antigen is associated with distant metastasis in lung cancer patients: immunohistochemical study on bronchofiberscopic biopsy specimens. Lung Cancer 20: 109–116. 9711529 10.1016/s0169-5002(98)00016-6

[6] Kim YJ, Borsig L, Varki NM, Varki A (1998) P-selectin deficiency attenuates tumor growth and metastasis. Proc Natl Acad Sci U S A 95: 9325–9330. 9689079 10.1073/pnas.95.16.9325 PMC21337

[7] Tatsumi M, Watanabe A, Sawada H, Yamada Y, Shino Y, et al (1998) Immunohistochemical expression of the sialyl Lewis x antigen on gastric cancer cells correlates with the presence of liver metastasis. Clin Exp Metastasis 16: 743–750. 10211987 10.1023/a:1006584829246

[8] Amado M, Carneiro F, Seixas M, Clausen H, Sobrinho-Simoes M (1998) Dimeric sialyl-Le(x) expression in gastric carcinoma correlates with venous invasion and poor outcome. Gastroenterology 114: 462–470. 9496936 10.1016/s0016-5085(98)70529-3

[9] Baldus SE, Zirbes TK, Monig SP, Engel S, Monaca E, et al (1998) Histopathological subtypes and prognosis of gastric cancer are correlated with the expression of mucin-associated sialylated antigens: Sialosyl-Lewis(a), Sialosyl-Lewis(x) and sialosyl-Tn. Tumour Biol 19: 445–453. 9817972 10.1159/000030036

[10] Grabowski P, Mann B, Mansmann U, Lovin N, Foss HD, et al (2000) Expression of SIALYL-Le(x) antigen defined by MAb AM-3 is an independent prognostic marker in colorectal carcinoma patients. Int J Cancer 88: 281–286. 11004681

[11] Nakamori S, Kameyama M, Imaoka S, Furukawa H, Ishikawa O, et al (1997) Involvement of carbohydrate antigen sialyl Lewis(x) in colorectal cancer metastasis. Dis Colon Rectum 40: 420–431. 9106690 10.1007/BF02258386

[12] Nakamori S, Nishihara S, Ikehara Y, Nagano H, Dono K, et al (1999) Molecular mechanism involved in increased expression of sialyl Lewis antigens in ductal carcinoma of the pancreas. J Exp Clin Cancer Res 18: 425–432. 10606190

[13] Harduin-Lepers A, Krzewinski-Recchi MA, Colomb F, Foulquier F, Groux-Degroote S, et al (2012) Sialyltransferases functions in cancers. Front Biosci (Elite Ed) 4: 499–515. 22201891 10.2741/e396

[14] Harduin-Lepers A, Mollicone R, Delannoy P, Oriol R (2005) The animal sialyltransferases and sialyltransferase-related genes: a phylogenetic approach. Glycobiology 15: 805–817. 15843597 10.1093/glycob/cwi063

[15] Harduin-Lepers A, Vallejo-Ruiz V, Krzewinski-Recchi MA, Samyn-Petit B, Julien S, et al (2001) The human sialyltransferase family. Biochimie 83: 727–737. 11530204 10.1016/s0300-9084(01)01301-3

[16] Kono M, Ohyama Y, Lee YC, Hamamoto T, Kojima N, et al (1997) Mouse beta-galactoside alpha 2,3-sialyltransferases: comparison of in vitro substrate specificities and tissue specific expression. Glycobiology 7: 469–479. 9184827 10.1093/glycob/7.4.469

[17] Okajima T, Fukumoto S, Miyazaki H, Ishida H, Kiso M, et al (1999) Molecular cloning of a novel alpha2,3-sialyltransferase (ST3Gal VI) that sialylates type II lactosamine structures on glycoproteins and glycolipids. J Biol Chem 274: 11479–11486. 10206952 10.1074/jbc.274.17.11479

[18] Ellies LG, Sperandio M, Underhill GH, Yousif J, Smith M, et al (2002) Sialyltransferase specificity in selectin ligand formation. Blood 100: 3618–3625. 12393657 10.1182/blood-2002-04-1007

[19] Sperandio M, Frommhold D, Babushkina I, Ellies LG, Olson TS, et al (2006) Alpha 2,3-sialyltransferase-IV is essential for L-selectin ligand function in inflammation. Eur J Immunol 36: 3207–3215. 17111351 10.1002/eji.200636157

[20] Kitagawa H, Paulson JC (1993) Cloning and expression of human Gal beta 1,3(4)GlcNAc alpha 2,3-sialyltransferase. Biochem Biophys Res Commun 194: 375–382. 8333853 10.1006/bbrc.1993.1830

[21] Colomb F, Krzewinski-Recchi MA, El Machhour F, Mensier E, Jaillard S, et al (2012) TNF regulates sialyl-Lewisx and 6-sulfo-sialyl-Lewisx expression in human lung through up-regulation of ST3GAL4 transcript isoform BX. Biochimie 94: 2045–2053. 22691873 10.1016/j.biochi.2012.05.030

[22] Carvalho AS, Harduin-Lepers A, Magalhaes A, Machado E, Mendes N, et al (2010) Differential expression of alpha-2,3-sialyltransferases and alpha-1,3/4-fucosyltransferases regulates the levels of sialyl Lewis a and sialyl Lewis x in gastrointestinal carcinoma cells. Int J Biochem Cell Biol 42: 80–89. 19781661 10.1016/j.biocel.2009.09.010

[23] Jun L, Yuanshu W, Yanying X, Zhongfa X, Jian Y, et al (2012) Altered mRNA expressions of sialyltransferases in human gastric cancer tissues. Med Oncol 29: 84–90. 21140242 10.1007/s12032-010-9771-1

[24] Petretti T, Schulze B, Schlag PM, Kemmner W (1999) Altered mRNA expression of glycosyltransferases in human gastric carcinomas. Biochim Biophys Acta 1428: 209–218. 10434038 10.1016/s0304-4165(99)00080-x

[25] Cazet A, Bobowski M, Rombouts Y, Lefebvre J, Steenackers A, et al (2012) The ganglioside G(D2) induces the constitutive activation of c-Met in MDA-MB-231 breast cancer cells expressing the G(D3) synthase. Glycobiology 22: 806–816. 22301273 10.1093/glycob/cws049

[26] Cazet A, Groux-Degroote S, Teylaert B, Kwon KM, Lehoux S, et al (2009) GD3 synthase overexpression enhances proliferation and migration of MDA-MB-231 breast cancer cells. Biol Chem 390: 601–609. 19335207 10.1515/BC.2009.054

[27] Cazet A, Julien S, Bobowski M, Krzewinski-Recchi MA, Harduin-Lepers A, et al (2010) Consequences of the expression of sialylated antigens in breast cancer. Carbohydr Res 345: 1377–1383. 20231016 10.1016/j.carres.2010.01.024

[28] Organ SL, Tsao MS (2011) An overview of the c-MET signaling pathway. Ther Adv Med Oncol 3: S7–S19. 22128289 10.1177/1758834011422556 PMC3225017

[29] Liu X, Newton RC, Scherle PA (2010) Developing c-MET pathway inhibitors for cancer therapy: progress and challenges. Trends Mol Med 16: 37–45. 20031486 10.1016/j.molmed.2009.11.005

[30] Drake PM, Cho W, Li B, Prakobphol A, Johansen E, et al (2010) Sweetening the pot: adding glycosylation to the biomarker discovery equation. Clin Chem 56: 223–236. 19959616 10.1373/clinchem.2009.136333 PMC2849286

[31] Hakomori S (2002) Glycosylation defining cancer malignancy: new wine in an old bottle. Proc Natl Acad Sci U S A 99: 10231–10233. 12149519 10.1073/pnas.172380699 PMC124893

[32] Dube DH, Bertozzi CR (2005) Glycans in cancer and inflammation--potential for therapeutics and diagnostics. Nat Rev Drug Discov 4: 477–488. 15931257 10.1038/nrd1751

[33] Varki A (2008) Sialic acids in human health and disease. Trends Mol Med 14: 351–360. 18606570 10.1016/j.molmed.2008.06.002 PMC2553044

[34] Kannagi R (1997) Carbohydrate-mediated cell adhesion involved in hematogenous metastasis of cancer. Glycoconj J 14: 577–584. 9298690 10.1023/a:1018532409041

[35] Takada A, Ohmori K, Yoneda T, Tsuyuoka K, Hasegawa A, et al (1993) Contribution of carbohydrate antigens sialyl Lewis A and sialyl Lewis X to adhesion of human cancer cells to vascular endothelium. Cancer Res 53: 354–361. 7678075

[36] Kannagi R, Izawa M, Koike T, Miyazaki K, Kimura N (2004) Carbohydrate-mediated cell adhesion in cancer metastasis and angiogenesis. Cancer Sci 95: 377–384. 15132763 10.1111/j.1349-7006.2004.tb03219.x PMC11159147

[37] Ichikawa D, Kitamura K, Tani N, Nishida S, Tsurutome H, et al (2000) Molecular detection of disseminated cancer cells in the peripheral blood and expression of sialylated antigens in colon cancers. J Surg Oncol 75: 98–102. 11064388 10.1002/1096-9098(200010)75:2<98::aid-jso5>3.0.co;2-r

[38] Nakamori S, Kameyama M, Imaoka S, Furukawa H, Ishikawa O, et al (1993) Increased expression of sialyl Lewisx antigen correlates with poor survival in patients with colorectal carcinoma: clinicopathological and immunohistochemical study. Cancer Res 53: 3632–3637. 8101764

[39] Aubert M, Panicot L, Crotte C, Gibier P, Lombardo D, et al (2000) Restoration of alpha(1,2) fucosyltransferase activity decreases adhesive and metastatic properties of human pancreatic cancer cells. Cancer Res 60: 1449–1456. 10728712

[40] Seales EC, Jurado GA, Brunson BA, Wakefield JK, Frost AR, et al (2005) Hypersialylation of beta1 integrins, observed in colon adenocarcinoma, may contribute to cancer progression by up-regulating cell motility. Cancer Res 65: 4645–4652. 15930282 10.1158/0008-5472.CAN-04-3117

[41] Seales EC, Shaikh FM, Woodard-Grice AV, Aggarwal P, McBrayer AC, et al (2005) A protein kinase C/Ras/ERK signaling pathway activates myeloid fibronectin receptors by altering beta1 integrin sialylation. J Biol Chem 280: 37610–37615. 16157583 10.1074/jbc.M508476200

[42] St Hill CA, Krieser K, Farooqui M (2011) Neutrophil interactions with sialyl Lewis X on human nonsmall cell lung carcinoma cells regulate invasive behavior. Cancer 117: 4493–4505. 21437888 10.1002/cncr.26059 PMC3134589

[43] Singh R, Subramanian S, Rhodes JM, Campbell BJ (2006) Peanut lectin stimulates proliferation of colon cancer cells by interaction with glycosylated CD44v6 isoforms and consequential activation of c-Met and MAPK: functional implications for disease-associated glycosylation changes. Glycobiology 16: 594–601. 16571666 10.1093/glycob/cwj108

[44] Smolen GA, Sordella R, Muir B, Mohapatra G, Barmettler A, et al (2006) Amplification of MET may identify a subset of cancers with extreme sensitivity to the selective tyrosine kinase inhibitor PHA-665752. Proc Natl Acad Sci U S A 103: 2316–2321. 16461907 10.1073/pnas.0508776103 PMC1413705

[45] Sierra JR, Tsao MS (2011) c-MET as a potential therapeutic target and biomarker in cancer. Ther Adv Med Oncol 3: S21–35. 22128285 10.1177/1758834011422557 PMC3225018

[46] Soman NR, Correa P, Ruiz BA, Wogan GN (1991) The TPR-MET oncogenic rearrangement is present and expressed in human gastric carcinoma and precursor lesions. Proc Natl Acad Sci U S A 88: 4892–4896. 2052572 10.1073/pnas.88.11.4892 PMC51773

[47] Yu J, Miehlke S, Ebert MP, Hoffmann J, Breidert M, et al (2000) Frequency of TPR-MET rearrangement in patients with gastric carcinoma and in first-degree relatives. Cancer 88: 1801–1806. 10760755

[48] Lee J, Seo JW, Jun HJ, Ki CS, Park SH, et al (2011) Impact of MET amplification on gastric cancer: possible roles as a novel prognostic marker and a potential therapeutic target. Oncol Rep 25: 1517–1524. 21424128 10.3892/or.2011.1219

[49] Dua R, Zhang J, Parry G, Penuel E (2011) Detection of hepatocyte growth factor (HGF) ligand-c-MET receptor activation in formalin-fixed paraffin embedded specimens by a novel proximity assay. PLoS One 6: e15932. 21283737 10.1371/journal.pone.0015932 PMC3024969

[50] Inoue T, Kataoka H, Goto K, Nagaike K, Igami K, et al (2004) Activation of c-Met (hepatocyte growth factor receptor) in human gastric cancer tissue. Cancer Sci 95: 803–808. 15504247 10.1111/j.1349-7006.2004.tb02185.x PMC11158965

[51] Lim SK, Choi YW, Lim IK, Park TJ (2012) BTG2 suppresses cancer cell migration through inhibition of Src-FAK signaling by downregulation of reactive oxygen species generation in mitochondria. Clin Exp Metastasis. 10.1007/s10585-012-9479-z 22562501

[52] Peng L, Ran YL, Hu H, Yu L, Liu Q, et al (2009) Secreted LOXL2 is a novel therapeutic target that promotes gastric cancer metastasis via the Src/FAK pathway. Carcinogenesis 30: 1660–1669. 19625348 10.1093/carcin/bgp178

[53] Sanchez-Bailon MP, Calcabrini A, Gomez-Dominguez D, Morte B, Martin-Forero E, et al (2012) Src kinases catalytic activity regulates proliferation, migration and invasiveness of MDA-MB-231 breast cancer cells. Cell Signal 24: 1276–1286. 22570868 10.1016/j.cellsig.2012.02.011

[54] Shen J, Xu L, Owonikoko TK, Sun SY, Khuri FR, et al (2012) NNK promotes migration and invasion of lung cancer cells through activation of c-Src/PKCiota/FAK loop. Cancer Lett 318: 106–113. 22178655 10.1016/j.canlet.2011.12.008 PMC3288633

[55] Birchmeier C, Birchmeier W, Gherardi E, Vande Woude GF (2003) Met, metastasis, motility and more. Nat Rev Mol Cell Biol 4: 915–925. 14685170 10.1038/nrm1261

[56] de Curtis I, Meldolesi J (2012) Cell surface dynamics - how Rho GTPases orchestrate the interplay between the plasma membrane and the cortical cytoskeleton. J Cell Sci. 10.1242/jcs.108266 23093576

[57] Machesky LM, Insall RH (1999) Signaling to actin dynamics. J Cell Biol 146: 267–272. 10427083 10.1083/jcb.146.2.267 PMC2156183

[58] Tapon N, Hall A (1997) Rho, Rac and Cdc42 GTPases regulate the organization of the actin cytoskeleton. Curr Opin Cell Biol 9: 86–92. 9013670 10.1016/s0955-0674(97)80156-1

[59] Hall A (1999) Signal transduction pathways regulated by the Rho family of small GTPases. Br J Cancer 80 Suppl 125–27. 10466757

[60] Kjoller L, Hall A (1999) Signaling to Rho GTPases. Exp Cell Res 253: 166–179. 10579921 10.1006/excr.1999.4674

[61] Pertz O (2010) Spatio-temporal Rho GTPase signaling - where are we now? J Cell Sci 123: 1841–1850. 20484664 10.1242/jcs.064345

[62] Evers EE, Zondag GC, Malliri A, Price LS, ten Klooster JP, et al (2000) Rho family proteins in cell adhesion and cell migration. Eur J Cancer 36: 1269–1274. 10882865 10.1016/s0959-8049(00)00091-5

[63] Parri M, Chiarugi P (2010) Rac and Rho GTPases in cancer cell motility control. Cell Commun Signal 8: 23. 20822528 10.1186/1478-811X-8-23 PMC2941746

[64] Wessler S, Gimona M, Rieder G (2011) Regulation of the actin cytoskeleton in Helicobacter pylori-induced migration and invasive growth of gastric epithelial cells. Cell Commun Signal 9: 27. 22044652 10.1186/1478-811X-9-27 PMC3214149

[65] Kamai T, Yamanishi T, Shirataki H, Takagi K, Asami H, et al (2004) Overexpression of RhoA, Rac1, and Cdc42 GTPases is associated with progression in testicular cancer. Clin Cancer Res 10: 4799–4805. 15269155 10.1158/1078-0432.CCR-0436-03

[66] Leve F, Marcondes TG, Bastos LG, Rabello SV, Tanaka MN, et al (2011) Lysophosphatidic acid induces a migratory phenotype through a crosstalk between RhoA-Rock and Src-FAK signalling in colon cancer cells. Eur J Pharmacol 671: 7–17. 21968138 10.1016/j.ejphar.2011.09.006

